# p53 activity contributes to defective interfollicular epidermal differentiation in hyperproliferative murine skin

**DOI:** 10.1111/bjd.14048

**Published:** 2015-11-20

**Authors:** D.L. Cottle, K. Kretzschmar, H.P. Gollnick, S.R. Quist

**Affiliations:** ^1^ Wellcome Trust–Medical Research Council Cambridge Stem Cell InstituteUniversity of CambridgeCambridge CB2 1QRU.K.; ^2^ Department of Biochemistry and Molecular BiologyMonash UniversityClayton 3800VICAustralia; ^3^ Centre for Stem Cell and Regenerative Medicine ResearchKing's College LondonLondon SE1 9RTU.K.; ^4^ Clinic of Dermatology and VenereologyOtto‐von‐Guericke UniversityDE‐39120MagdeburgGermany; ^5^ CR‐UK Cambridge Research InstituteLi Ka Shing CentreCambridge CB2 0REU.K.; ^6^ Present address: Hubrecht Institute–KNAW and University Medical CentreUtrecht Uppsalalaan 83584 CTUtrechtthe Netherlands

dear editor, Skin diseases affect a significant percentage of the population and are often the result of a complex interplay between autoimmune dysregulation, and abnormal epidermal differentiation and proliferation. Origins may be genetic and/or environmental, and while no complete cure exists for conditions such as psoriasis, a range of treatments, including retinoids and antibodies against tumour necrosis factor‐α and interleukin‐17, have shown therapeutic efficacy, although relapses can occur.^1^ While there is no definitive mouse model for psoriasis, some do present with abnormal skin phenotypes that have revealed interesting molecular drivers.^1^ For example, agonist‐treated peroxisome proliferator‐activated receptor (PPAR) β/δ transgenic mice develop psoriasis‐like conditions with failed compaction of the granular layer, which can be countered by PPARβ/δ antagonists.^2^ PPARβ/δ is also upregulated in human psoriasis and murine ichthyosis,^2^, ^3^ but has proinflammatory, anti‐inflammatory and prodifferentiation properties in various contexts.^4^, ^5^ PPARγ, although predominantly expressed in the sebaceous gland, has similar prodifferentiation effects, and can reduce inflammation and promote barrier formation in mice with induced parakeratosis (Fig. S1; see Supporting Information).^6^

The tumour suppressor p53 is upregulated in the pathogenesis of human chronic plaque‐type psoriasis (Fig. 1a), and its role in skin disease has long been questioned.^7^ In additional animal studies, p53 has been found to be largely dispensable to epidermal homeostasis, with gene loss only causing minor alterations in murine catagen,^8^ and paradoxically, *p53* deletion reduces oncogenesis in transgenic mouse skin carcinogenesis studies.^9^ *p53* knockdown also promotes squamous differentiation in human keratinocytes cultured *in vitro*,^10^ which suggests p53 activation may impair keratinocyte differentiation in the interfollicular epidermis; however, this has not been tested *in vivo*.

**Figure 1:**
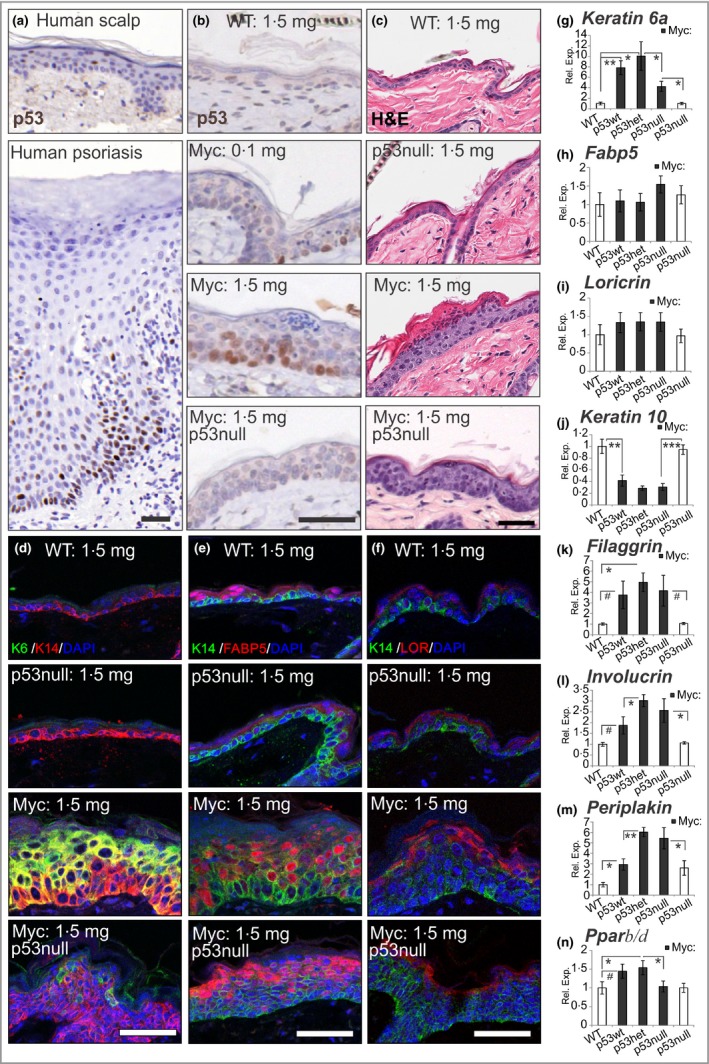
Characterization of *K14MycER p53null* mouse epidermis. (a) Human scalp and psoriasis lesions immunostained for p53 (*n* = 6). (b) Mouse back skin from wild‐type (WT:), *K14MycER* (Myc:) and *K14MycER p53* knockout (Myc: p53null) mice 4 days after treatment with 0·1 mg or 1·5 mg 4‐hydroxytamoxifen as indicated, immunostained for p53 and counterstained with haematoxylin. (c) Mice treated as in (b), including p53 knockout (p53null) controls stained with haematoxylin and eosin (H&E). The granular layer was not visible in 21·39 ± 0·01% of *K14MycER p53* wild‐type (WT;* p53wt*) skin (as a proportion of length) and 22·64 ± 0·03% of *K14MycER p53* heterozygous (*p53het*) skin. In contrast, the granular layer was only absent in 10·85 ± 0·03% of *K14MycER p53null* skin (*P < 0·05). (d) Mouse skin immunostained for keratin 6 (K6) and keratin 14 (K14), and counterstained with nuclear dye 4′,6‐diamidino‐2‐phenylindole (DAPI); (e) mouse skin immunostained for K14, fatty acid binding protein 5 (FABP5) and DAPI; and (f) mouse skin immunostained for K14, loricrin (LOR) and DAPI. (g–n) Quantitative reverse transcription polymerase chain reaction for indicated mRNAs relative to *Gapdh,* standardized to WT mice (defined as 1). *K14MycER* (Myc:) mice shown by grey bars of p53wt, p53het and p53null status. (j) MYC activity alone induced downregulation of *Krt10* mRNA, although (k) the K10 protein persists in this time frame and there is upregulation of *Flg* mRNA (Fig. S1; see Supporting Information). (l, m) Genes that showed MYC and p53‐dependent regulation included *Ivl* and *Ppl*, such that deletion of even one functional *p53* allele resulted in a significant upregulation of mRNA. (g, n) Most significantly, hyperproliferative *Krt6a* and *Ppar*β*/*δ expression, which was upregulated in *K14MycER p53wt/het* mice, was reduced in *K14MycER p53null* mice. We have shown previously that *K14MycER p53null* mice have increased *Pparg mRNA* expression,^13^ and here demonstrate the increased peroxisome proliferator‐activated receptor (PPAR) γ protein expression is predominantly in the sebaceous gland (Fig. S1; see Supporting Information). *n* = 3–5. Error bars represent SEM. ^#^ *P* < 0·06; *P < 0·05, **P < 0·01, ***P < 0·005. Scale bars 50 μm. Rel. Exp., relative expression.

A parakeratotic differentiation programme can be invoked by high MYC activity in keratinocytes; thus, *K14MycER* mice form a useful model of hyperproliferative skin. They overexpress MYC fused with the tamoxifen‐responsive mutant oestrogen receptor ligand binding domain in the keratin 14 (K14)‐positive basal layer of the epidermis and, upon activation with high‐dose 4‐hydroxytamoxifen (4OHT), exhibit parakeratotic lesions of acanthosis, hyperkeratosis and dermal inflammatory infiltration (see Fig. S1; see Supporting Information).^11^, ^12^ Our *K14MycER* mice also show dose‐dependent activation of the tumour suppressor p53 (Fig. 1b).^13^

We previously crossed *K14MycER* mice with *p53* knockout animals and demonstrated that aberrant p53 activity interferes with sebaceous gland differentiation by impairing androgen receptor function.^13^ In this study we investigated if p53 activity also contributes to defective interfollicular epidermal differentiation in the same cohort of animals. Full materials and methods are available in Appendix S1 (see Supporting Information). Our results show *K14MycER p53null* mice exhibited persistent hyperproliferation (Fig. S1; see Supporting Information),^13^ skin thickening and K14‐positive basal layer expansion (Fig. 1c, d); however, deletion of *p53* causes a number of positive changes, with reduced keratin 6 (K6) expression (Fig. 1d, g), partial redistribution of the keratinocyte differentiation marker fatty acid binding protein 5 (FABP5) towards terminal differentiating layers (Fig. 1e, h), and improved granular layer compaction (Fig. 1). Additional quantitative reverse‐transcription polymerase chain reaction (qRT‐PCR) analysis showed that MYC activity reduced *Krt10* mRNA expression (Fig. 1j), although keratin 10 (K10) protein persisted in the uppermost keratinocyte layers (Fig. S1; see Supporting Information). MYC activity also promoted the expression of *Flg* and *Ivl* but these (and *K10*) did not change further upon deletion of *p53* (Fig. 1j–m). However, loss of *p53* reduced *Krt6a* (Fig. 1g), upregulated *Pparg* and normalized mRNA expression of *Pparb/d* [Fig. 1n; Fig. S1 (see Supporting Information)].^13^

Retinoic acid (RA) signalling is important to skin biology yet there is no previous evidence for cross‐talk between p53 and RA signalling in skin. A recent study has demonstrated that p21(RAC)‐activated kinase 2 (PAK2)‐phosphorylated MYC binds and co‐activates RA receptor (RAR) α, while unphosphorylated MYC acts as a co‐repressor.^14^ As we observed nonapoptotic *p53* activation in response to MYC, we set out to examine if this was related to RAR signalling by using the reverse agonist BMS493 to promote stabilization of RAR/retinoid X receptor repressive complexes.^15^ This drug promoted granular formation and prevented the induction of *p53* (Fig. 2a, b). We had also previously generated *K14MycAER* mice, possessing three MYC point mutations to prevent PAK2 phosphorylation, and predicted ‘MYCA’ would therefore mimic BMS493 to function as a RAR co‐repressor. Our prediction proved true, as *K14MycAER* mice did, indeed, retain granular formation (Fig. 2c), and showed markedly reduced *p53* expression and p53 activation (Fig. 2d, e). As in *K14MycER* mice, hyperproliferation, skin thickening and basal layer expansion still occurred [Fig. 2c, f; Fig. S1 (see Supporting Information)], and *Krt10* mRNA expression was also reduced, while the K10 protein persisted [Fig. 2i; Fig. S1 (see Supporting Information)]. However, points of difference included that K6 was not as greatly upregulated and FABP5 was predominantly observed in upper differentiating keratinocytes in *K14MycAER* mice (Fig. 2f, g). Loricrin was also more compacted (Fig. 2h). Further qRT‐PCR analysis of *K14MycAER* mice confirmed reduced *Krt6a* expression (Fig. 2j), along with upregulation of *Pparg* and normalized *Pparb/d* mRNA expression (Fig. 2k, l). Thus, *K14MycAER* mice phenocopy *K14MycER p53null* mice (Fig. 2m). Of further interest, in contrast to *K14MycER* mice, *K14MycAER* mice also maintained their granular layer when challenged with the strongly p53‐activating compound, camptothecin (Fig. S1; see Supporting Information), suggesting PAK2 unphosphorylated MYC is the form of MYC that counteracts p53.

**Figure 2:**
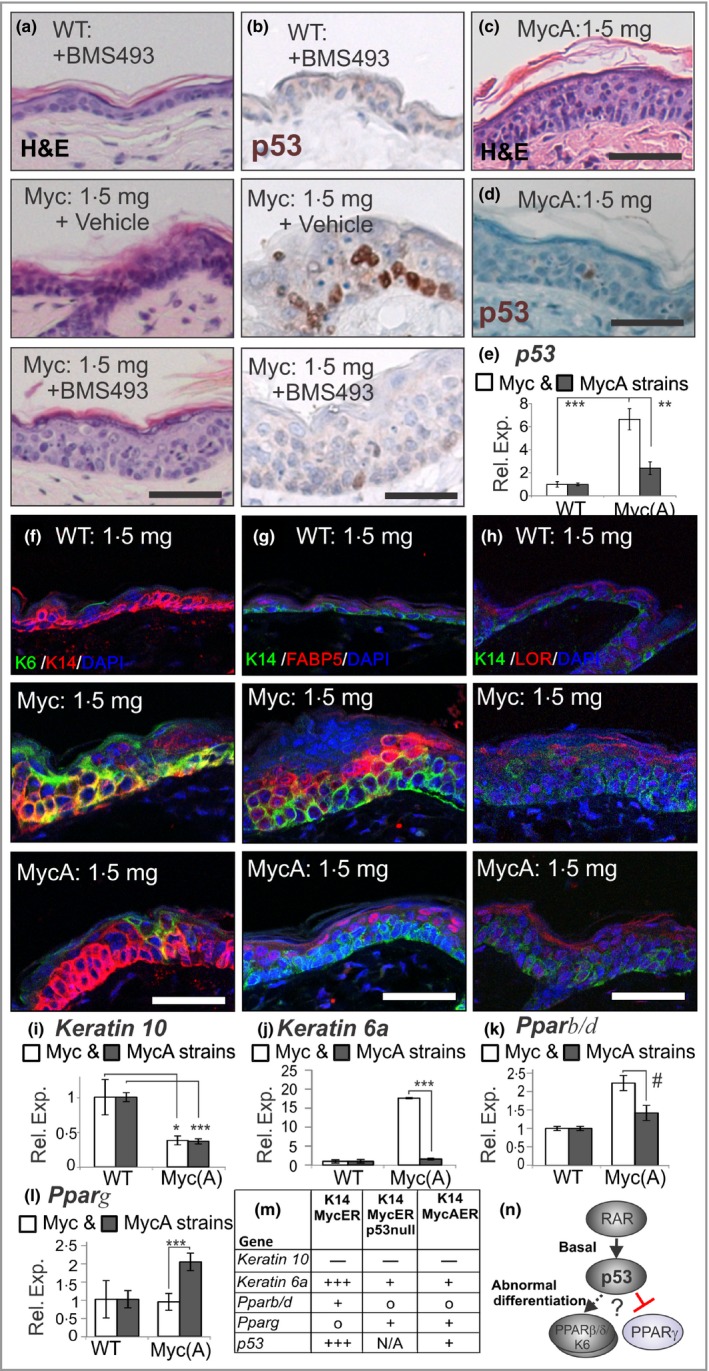
Retinoic acid signalling and p53. (a) Mouse telogen back skin [wild‐type (WT:) and *K14MycER* (Myc:) mice] treated once with acetone or 1·5 mg 4‐hydroxytamoxifen (4OHT) and daily with 0·016 mg BMS493 for 4 days, stained with haematoxylin and eosin (H&E) and (b) immunostained for p53 with haematoxylin counterstain. (c) Haematoxylin and eosin analysis of *K14MycAER* (MycA:) mice treated with 1·5 mg 4OHT after 4 days. (d) p53 immunostaining of mice in (c) with haematoxylin counterstain. (e) Quantitative reverse transcription polymerase chain reaction (qRT‐PCR) for *p53* mRNA relative to *Gapdh,* standardized to wild‐type (WT) mice (defined as 1). Mice (including transgenics and WT siblings) from the *K14MycER* (Myc:) strain are shown by white bars and mice from the *K14MycAER* (MycA:) strains are shown by the grey bars. (f) Immunostained for keratin 6 (K6), keratin 14 (K14) and 4′,6‐diamidino‐2‐phenylindole (DAPI), (g) immunostained for K14, fatty acid binding protein 5 (FABP5) and DAPI, and (h) immunostained for K14, loricrin (LOR) and DAPI. (i–l) qRT‐PCR for indicated mRNAs relative to *Gapdh,* standardized to WT mice (defined as 1). Mice (including transgenics and WT siblings) from the *K14MycER* (Myc:) strain are shown by white bars and mice from the *K14MycAER* (MycA:) strains are shown by the grey bars. (m) Summary of gene changes in transgenic mouse strains. (+) indicates gain, (−) indicates reduction, (o) indicates unchanged. Magnitude indicated by number of ± symbols. (o) Summary model of proposed signalling pathway (note that BMS493 and MYCA can inhibit retinoic acid receptor activity and prevent p53 activity). *n* = 3–9. Error bars represent SEM. ^#^ *P* < 0·06, *P < 0·05, ***P < 0·005, **P < 0·01. Scale bars = 50 μm. N/A, not applicable; Rel. Exp., relative expression; PPAR, peroxisome proliferator‐activated receptor.

A question that arises is how p53 activity predominantly detected in basal cells can trigger such changes in differentiating keratinocytes. One possibility is that p53 promotes basal keratinocyte‐secreted inflammatory mediators that influence differentiating cells. In line with this general idea, we recently showed how chemokine upregulation increases disease severity by impairing keratinocyte terminal differentiation in a mouse model of harlequin ichthyosis.^3^

In conclusion, we identified that p53 signalling reduces *Pparg* and promotes *Pparb/d* and *Krt6a* expression. This has physiological significance as it controls granular layer formation (Fig. 2n). We also highlight for the first time, the significant convergence of the p53 and RAR signalling pathways via MYC and demonstrate that repression of RARs can inhibit p53 activity to restore granular layer formation, which is presumably good for barrier function. Given p53 is an integrator of multiple stress responses, these findings may provide mechanistic insight into the pathogenesis of human skin diseases where granular formation is disrupted.

## Supporting information


**Appendix S1**. Materials and methods.
**Fig S1**. Further characterization of *K14MycER, K14MycER p53null* and *K14MycAER* mouse epidermis.
