# Supplementary material for: p53 activity contributes to defective interfollicular epidermal differentiation in hyperproliferative murine skin
Source: Br J Dermatol. 2015 Nov 20;174(1):204–8. doi: 10.1111/bjd.14048 (PMC4832295; doi:10.1111/bjd.14048)

**Supporting Material**

**Materials and methods:**

*Ethics Statement:* All human tissue samples were taken with informed consent and processed in accordance with local ethics committees, CR-UK Cambridge Research Institute (number 08/H0306/30), University of Magdeburg and the German Medical Council. Mouse experiments are subject to Cancer Research (CR)-UK ethical review and performed under the terms of a UK Government Home Office licence.

*Transgenic mice:* Mice were maintained on a B6CBAF1 background. *K14MycER* transgenic mouse 2184C.1 (*K14MycER*) [^1^](#_ENREF_1) and *K14MycAER* 6972.C3 (*K14MycAER*), express similar levels of MYC transgene protein [^2^](#_ENREF_2) and are presented in this study. *K14MycER* *p53null* mice and experimental design has been described previously [^3^](#_ENREF_3).

*Topical Application:* Mice were treated with 4-hydroxytamoxifen (4OHT) and Camptothecin chemical compounds as previously described[^3^](#_ENREF_3). For retinoids, mice received daily doses of 100 μL of acetone and/or 16 μg BMS493 (Sigma-Aldrich, catalogue B6688) in 100 μL acetone for 4 days (similar to a protocol described previously[^4^](#_ENREF_4)), in addition to one dose of 1.5mg 4OHT at Day 0.

*Antibodies and Microscopy:* Antibodies, immunofluorescence (IF), semi-automated immunohistochemistry (IHC) using Ventana Discovery system, and microscopy was performed as described previously [^3^](#_ENREF_3)^,^[^5^](#_ENREF_5). Antibodies include: goat anti-Fatty Acid Binding Protein 5 (FABP5)-biotin R&D Systems, MN USA catalogue BAF1476, IF 1:50; rabbit anti mouse-Keratin 6 (K6) Covance, NJ USA, catalogue PRB-169P, IHC 1:250, IF 1:500; rabbit anti mouse-Keratin 10 (K10) Covance, NJ USA, catalogue PRB-159P, IF 1:500; mouse anti-Keratin 14 (K14) in house Clone LL002 IF 1:250; rabbit anti-Ki67 (SP6) Abcam, Cambridge UK, catalogue ab16667, IF 1:100; rabbit anti-Loricrin (LOR) Covance, NJ USA, catalogue PRB-145P, IHC 1:500; rabbit anti-p53 CM5 Novocastra Reagents-Leica Biosystems, Milton Keynes UK, catalogue p53-CM5p, IHC 1:200; rabbit anti-PPARdelta LifeSpan Biosciences Inc, WA USA, catalogue LS-B45, IF 1:50; rabbit anti-PPARgamma Cell Signaling Technologies, MA USA, catalogue 2435S, IF 1:100. Secondary antibodies and streptavidin were from Molecular Probes, Invitrogen, Paisley UK.

*qRT-PCR:*  Whole back skin RNA preparations were processed and qRT-PCR analysis were performed as described elsewhere [^3^](#_ENREF_3). Taqman probes were *Ppard (Pparb)* Mm01305434_m1, *Pparg *Mm01184322_m1, *Keratin 6a* Mm00833464_g1, *Keratin 10* Mm03009921_m1, *p53* Mm01337166_mH, *Fabp5* Mm00783731_s1, *Involucrin* Mm00515219_m1, *Filaggrin* Mm01716522_m1, *Loricrin* Mm01219285_m1, *Periplakin* Mm00447206_m1 and *Gapdh* 4352932-1102038.

*Quantification and Statistics:* Quantification of granular layer loss as a proportion of skin length, was determined from at least 15 images of 10x magnification H&E stained tissue sections. Vertically sectioned regions were examined, with severely scabbed and wounded regions excluded from analysis. Statistical analysis was performed using the unpaired Student’s t-test.

**Additional Results:**

H&E analysis of *K14MycER, K14MycER p53null* and *K14MycAER* mice compared with *Wild type* (WT) and *p53null* mice, treated with 1x1.5mg 4OHT after 4 days, showed an increase number of cells in the dermis of all MYC/MYCA activated mice (regardless of p53 status), suggestive of inflammatory infiltrates (Fig. S1A). Hyperproliferation was also evident in treated *K14MycER, K14MycER p53null* and *K14MycAER* mice compared with *Wild type* (WT) and *p53null* mice, as shown by increased detection of Ki67+ve keratinocytes in the IFE (Fig. S1b).

Despite the dramatic down-regulation of *K10* mRNA observed upon MYC-activation, K10 protein was still detectable in *K14MycER, K14MycER p53null* and *K14MycAER* mice by IHC-IF, 4 days following treatment with 1x1.5mg (Fig. S1c), although only the upper most layers were K10+ve in these mice, while the first suprabasal layer was K10+ve in WT animals. Thus the K10 down-regulation observed was more likely a consequence of disproportional expansion of K14-expressing (K10-negative, K6-positive, FABP5-positive) cells in whole cell preparations.

Up-regulation of *Pparg* mRNA was previously described[^3^](#_ENREF_3) for *K14MycER p53null* mice and was also observed in *K14MycAER* but not *K14MycER* mice (Fig. 1l). Analysis of PPARγ expression by immunostaining of skin sections showed the predominant expression was in the nuclei of differentiating sebocytes and the increased *Pparg* mRNA detected from *K14MycER p53null* and *K14MycAER* mice likely reflects the increased numbers of differentiating sebocytes in these mice. However, a very weak signal throughout the epidermis and slightly higher signal in the granular layer was also observed, so PPARγ may also contribute to IFE differentiation (Fig. S1d).

Up-regulation of *Ppard* mRNA was detected in *K14MycER* mice but not *K14MycER p53null,* nor *K14MycAER* mice (Fig. 1n, 2k). Analysis of PPAR expression by immunostaining showed PPAR in the nuclei of most keratinocytes and the granular layer in all samples. However in *K14MycER* mice, consistent with mRNA upregulation, PPAR showed acquisition of a strong cytoplasmic pool of PPAR. This cytoplasmic pool of PPAR was also acquired in *K14MycAER* and *K14MycER p53null* mice but a lesser extent (Fig. S1e). As PPAR up-regulation is associated with human psoriasis and PPAR activity in transgenic mice causes psoriasis-like skin lesions, the differential regulation of *Ppard* mRNA and PPAR protein in these models likely contributes to the phenotypic differences observed.

Camptothecin (Campto), a DNA-damaging agent, is a well-established activator of p53 and had been previously applied topically to mouse skin [^6^](#_ENREF_6)^,^[^7^](#_ENREF_7) and in contrast to UVB irradiation, activates p53 in a high number of epidermal cells. We then challenged *K14MycAER* and *K14MycER* mice with Camptothecin, to force p53 activation on a background of low-dose 0.1 mg 4OHT. DMSO was included in the acetone vehicle to promote uptake of insoluble Camptothecin. Low dose 4OHT was selected such that p53-activity would be mostly attributable to Camptothecin, however the presence of DMSO increased the activity of low-dose 4OHT over the same dose in pure acetone, but not to the same extent as 1.5 mg 4OHT. Camptothecin did induce some background apoptosis as determined from cleaved capase-3 staining but given the apoptosis-resistant nature of skin, this was at fairly low levels [^3^](#_ENREF_3). As expected low-dose 4OHT *K14MycER* mice treated with Camptothecin, showed more disruption of the granular layer, while *K14MycAER* mice treated with Camptothecin continued to resist granular layer destruction (Fig. S1f,g). This suggests the PAK2-unphosphorylated form of MYC is the form of MYC with inhibits p53 function. Of interest, Camptothecin treatment appeared to alter the basal layer morphology to appear the same as suprabasal layers, suggesting Camptothecin forced terminal albeit abnormal differentiation.

**Acknowledgements:** We thank Fiona M. Watt for funding this study via grants from the MRC, Wellcome Trust, CRUK, EU FP7 programme, the University of Cambridge and Hutchison Whampoa Ltd. KK was the recipient of MRC PhD studentship. We also thank the CRI and SCI core staff, and the Monash Histology Platform for expert technical assistance. We thank Ian M. Smyth for advice and resources to finalise this project.

**Author Contributions:** DLC conceived the project, performed the experiments and wrote the manuscript. KK assisted with experimental analysis. HPG and SRQ collected human patient biopsies for analysis. KK, HPG and SRQ all provided critical feedback on manuscript drafts.

**Supporting Figure Legend:**

a) Mouse back skin from wild-type (WT:), *K14MycER* (Myc:), and *K14MycER* *p53 knock-out* (Myc: p53null), *K14MycAER* (MycA:), and p53 knock-out (p53null) mice, 4 days following 1.5 mg 4-hydroxytamoxifen (4OHT) treatment stained with haematoxylin & eosin (H&E). Note the increase in dermal cells following MYC/MYCA activation. b) Mouse skin immunostained for Ki67, KERATIN 14 (K14), and counter-stained with nuclear dye 4',6-diamidino-2-phenylindole (DAPI), c) Mouse skin immunostained for KERATIN 10 (K10), K14 and DAPI, d) Mouse skin immunostained for PPARγ, K14 and DAPI, e) Mouse skin immunostained for PPAR, K14 and DAPI, f) *K14MycER* (Myc:) and *K14MycAER* (MycA:) mice treated once with 0.1 mg 4OHT and daily with 1 mg of Camptothecin (Campto) or DMSO/acetone vehicle for 4 days, stained for H&E. g) Quantification of mice in (f) for the percentage of epidermis by length, without a granular layer in H&E cross-sections. *K14MycER* (Myc) mice shown in white bars and *K14MycAER* (MycA) mice are shown in grey bars. n=3-9. Error bars represent SEM. *p<0.05, ***p<0.005, **p<0.01. NS=not significant. Scale bars 50 μm.

**References for Supporting Material**

1 Arnold I, Watt FM. c-Myc activation in transgenic mouse epidermis results in mobilization of stem cells and differentiation of their progeny. *Curr Biol* 2001; **11**: 558-68.

2 Berta MA, Baker CM, Cottle DL *et al.* Dose and context dependent effects of Myc on epidermal stem cell proliferation and differentiation. *EMBO Mol Med* 2010; **2**: 16-25.

3 Cottle DL, Kretzschmar K, Schweiger PJ *et al.* c-MYC-Induced Sebaceous Gland Differentiation Is Controlled by an Androgen Receptor/p53 Axis. *Cell Rep* 2013; **3**: 427-41.

4 Chapellier B, Mark M, Messaddeq N *et al.* Physiological and retinoid-induced proliferations of epidermis basal keratinocytes are differently controlled. *The EMBO journal* 2002; **21**: 3402-13.

5 Cottle DL, Ursino GM, Ip SC *et al.* Fetal inhibition of inflammation improves disease phenotypes in harlequin ichthyosis. *Hum Mol Genet* 2014.

6 Gao XM, Perchellet EM, Davis AW *et al.* Camptothecin post-treatments inhibit the biochemical events linked to the tumor-promoting component of carcinogenesis in mouse epidermis in vivo. *International journal of cancer. Journal international du cancer* 1996; **66**: 496-505.

7 Gao XM, Perchellet EM, Davis AW *et al.* Characterization of the antitumor-promoting activity of camptothecin in SENCAR mouse skin. *Carcinogenesis* 1996; **17**: 1141-8.


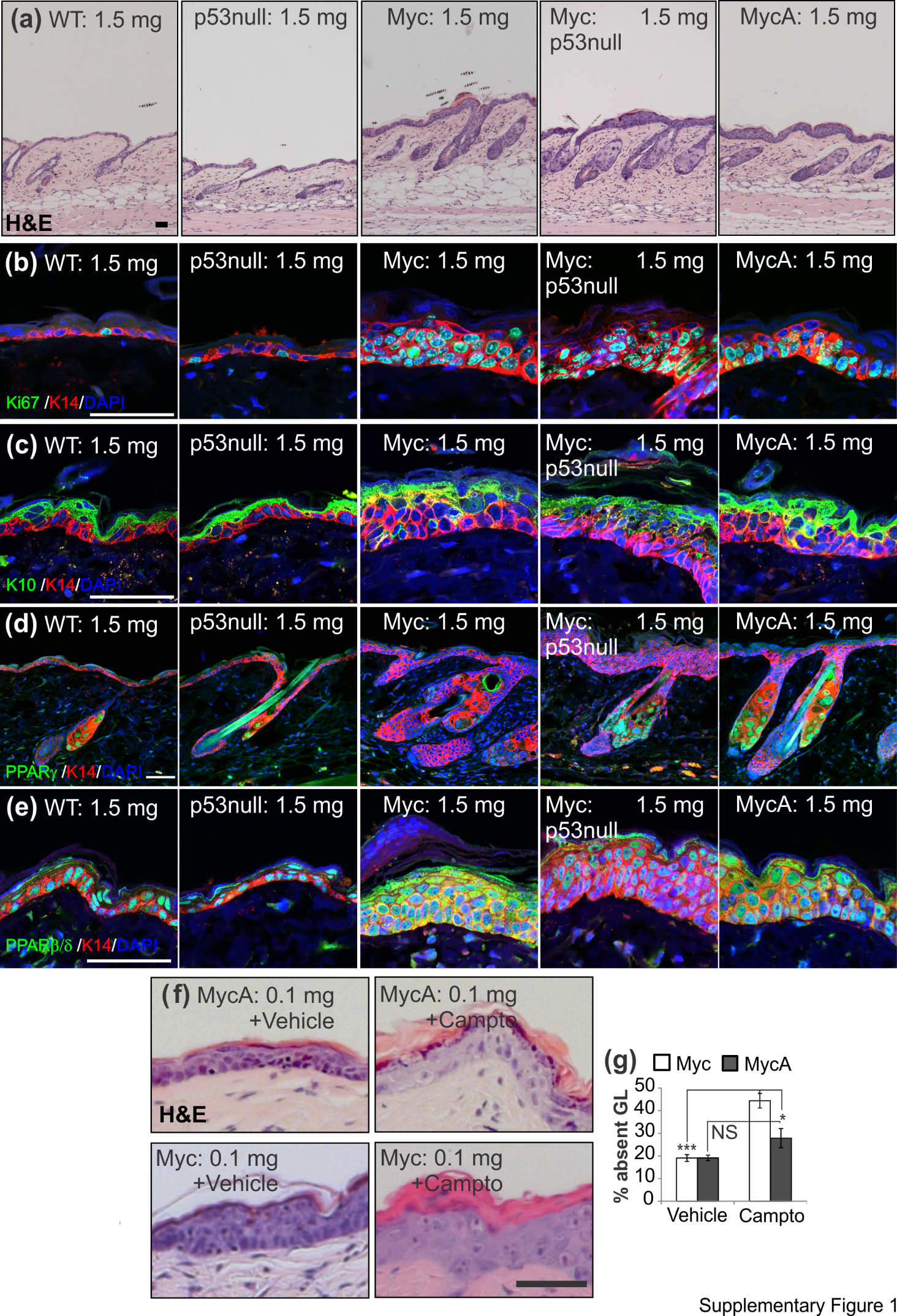

Supplement: Supplementary file 1 — Appendix S1. Materials and methods. Fig S1. Further characterization of K14MycER, K14MycER p53null and K14MycAER mouse epidermis. [file BJD-174-204-s001.docx]
